# Effect of an early music intervention on emotional and neurodevelopmental outcomes of preterm infants at 12 and 24 months

**DOI:** 10.3389/fpsyg.2024.1443080

**Published:** 2024-10-21

**Authors:** Manuela Filippa, Lara Lordier, Fleur Lejeune, Joana Sa De Almeida, Petra Susan Hüppi, Francisca Barcos-Munoz, Maria Grazia Monaci, Cristina Borradori-Tolsa

**Affiliations:** ^1^Department of Psychology and Educational Sciences, University of Geneva, Geneva, Switzerland; ^2^Department of Pediatrics, Obstetrics and Gynaecology, Division of Development and Growth, University of Geneva, Geneva, Switzerland; ^3^Department of Psychology, University of Aosta Valley, Aosta, Italy

**Keywords:** preterm infants, music intervention, emotion, attention, effortful control

## Abstract

**Background:**

Few studies have found long-term effects of early musical environmental enrichment in the NICU on preterm infant’s development. This study examines how early music enrichment affects emotional development and effortful control abilities in 12- and 24-month-old very preterm (VPT) infants.

**Methods:**

One hundred nineteen newborns were recruited, including 83 VPTs and 36 full-term (FT) infants. The VPT infants were randomly assigned to the music intervention (44 VPT-Music) or control (39 VPT-control) groups. VPT-Music infants listened specifically designed music intervention from the 33rd week of gestation until hospital discharge. At 12 and 24 months, children were clinically evaluated using the Bayley-III Scales of Infant and Toddler Development and the Laboratory Temperament Assessment Battery, and at 24 months, with 3 additional episodes of the Effortful Control Battery.

**Results and discussion:**

Our analysis showed that during a fear eliciting task, the VPT-Music group expressed lower level of fear reactivity and higher positive motor actions than VPT-controls and FT infants. At 24 months, the VPT-music group had lower scores for negative motor actions in the joy task, compared to both VPT-control and FT groups. In addition, both FT and VPT-music had higher scores of sustained attention compared to VPT-controls, but the contrasts were not significant. No significant effects on mental, language and motor outcomes were identified and for all three dimensions of the ECBQ.

**Conclusion:**

The present study suggests that an early music intervention in the NICU might influence preterm children’s emotional processing at 12 and 24 months. Limitations and suggestions for future research are highlighted.

## 1 Introduction

Remarkable advances in obstetric management and perinatal care have substantially increased the survival rates of preterm neonates, particularly those delivered at the earliest gestational ages (Chen et al., 2016; Pierrat et al., 2017). However, this commendable increase in survival is accompanied with a range of health sequelae and neurodevelopmental challenges, including motor and cognitive impairments, as well as behavioral difficulties, spanning from early infancy to adulthood (Arpi and Ferrari, 2013; Bhutta et al., 2002; Johnson and Marlow, 2017; Montagna and Nosarti, 2016; Ritchie et al., 2015; Twilhaar et al., 2018). Notably, during childhood, a distinctive “premature behavioral phenotype” emerges, typified by cardinal features encompassing symptoms of emotional lability social problems, as well as an increased risk of presenting *attention deficit and hyperactivity disorder*, autism spectrum disorder and anxiety disorders, in comparison to full-term newborns (Treyvaud et al., 2014).

Emphasis has recently been focused on the effects of prematurity on socio-emotional development, with difficulties spanning from infancy (Clark et al., 2008; Langerock et al., 2013; Klein et al., 2013), childhood (Gardner et al., 2004; Ritchie et al., 2015; Treyvaud et al., 2013) and extending into adolescence (Healy et al., 2013).

In a meta-analysis, it has been proposed that preterm children exhibit a temperament that is less well-regulated than that of full-term infants (Cassiano et al., 2020): in fact, they exhibit a higher level of activity, a lower attentional focus compared to their full-term peer group.

Moreover, compared to their full-term peers, very premature children (VPT) (born before 32 weeks’ gestation) show increased difficulties in controlling fear and anger in the early years (Langerock et al., 2013; Lejeune et al., 2015; Witt et al., 2014), as well as difficulties in recognizing emotional content and regulating social behavior at school age (Lejeune et al., 2016).

In the study conducted by Langerock et al. (2013) forty-one VPT infants were compared with a full-term born control group utilizing a standardized behavioral assessment, the Laboratory Temperament Assessment Battery (Lab-TAB) (Goldsmith and Rothbart, 1993). This assessment aimed to measure emotional reactivity across joy, anger and fear-inducing stimuli, alongside measuring sustained attention. Analysis of emotional manifestations and emotional regulation strategies indicated that while VPT infants exhibited comparable levels of joy expression to their full-term counterparts during joy-evoking scenarios, they displayed significantly heightened reactivity in anger-provoking situations and attenuated responsiveness in fear-evoking contexts by the age of 12 months-old. Notably, across all three emotion-eliciting situations, VPT reacted with a higher level of motor activity, indicating a high level of arousal in response to stimulation. Even if longitudinal studies are needed to disentangle this specific correlation (Joseph et al., 2023), anger and attention difficulties in 12-month-old VPT infants can be early indicators of ADHD due to their critical role in emotional and cognitive development. As ADHD involves deficits in executive functions, including working memory, cognitive flexibility, and inhibitory control, infants who show early difficulties in regulating negative emotions and problems with maintaining attention, shifting focus, and processing information are considered at risk (Stephens et al., 2021). Longitudinal trajectories of emotional regulation and inhibitory capacities were investigated (Montagna and Nosarti, 2016). Specifically, one study (Lejeune et al., 2015) was conducted using the Early Childhood Behavior Questionnaire (ECBQ) filled by parents (Putnam et al., 2006) alongside three episodes derived from the Effortful Control Battery, which evaluate two components of effortful control: delaying (waiting for a pleasant event) and inhibiting or initiating activity to signal (taking turns), respectively. VPT children were described by parents as more anxious and hypersensitive than full-term controls. This profile could be linked to later internalizing problems frequently reported in VPT children (Johnson and Marlow, 2011). Greater difficulties in maintaining inhibitory control during the effortful control tasks are also observed in VPT children (Taylor et al., 2023), especially at early school age (Réveillon et al., 2018). Witt et al. (2014) evaluated VPT children at 42 months of age with a series of specific neuropsychological tests. VPT children showed difficulties in labeling emotions and greater reactivity to fear and frustration, as well as difficulties in inhibition. These studies highlight that VPT infants exhibit heightened emotional reactivity, difficulties in inhibitory control and increased motor activity, all of which have repercussions on behavioral development and long-term social adaptation. Indeed, the early development of appropriate behavioral strategies for emotional regulation has been shown to be one of the main predictors of emotional (Berking and Wupperman, 2012), cognitive (Cisler and Olatunji, 2012) and social adaptation later in life.

The underlying alterations in brain development that lead to the high percentage of adverse outcome are not yet fully understood. However, as recent literature suggests, a major cause appears to be a combination of prenatal conditions leading to premature birth, postnatal brain injury, particularly white matter injury and impaired brain maturation (Inder et al., 2023; Miller et al., 2005; Volpe, 2019). It has been well demonstrated that the brain develops during the first 6 months of fetal life mainly under the influence of genetic influences, which are then replaced by intra-uterine environmental factors or external factors in the neonatal intensive care units (with related stressful events) that can alter the typical development of brain structures, leading to subsequent functional consequences (Cheong et al., 2020; Lammertink et al., 2021; McGowan and Vohr, 2019; Vasung et al., 2019). Indeed, premature birth generally entails a radical change of environment for the infant depriving him or her of the expected intra-uterine factors and exposing him or her to a variety of stressful sensory stimuli and procedures in hospital wards: equipment, pain, mobilization, noise. Parents of children born prematurely are also exposed to high levels of stress, anxiety and depression (Treyvaud et al., 2014). These multifaceted factors collectively shape maternal sensitivity and responsiveness during interactions with their infants, thereby exerting a profound impact on the child’s developmental trajectory (Forcada-Guex et al., 2011), with enduring ramifications for their socio-emotional competencies (Turpin et al., 2019).

In recent decades, research has provided a better understanding of the complex relationships existing between the environment and brain development (Tooley et al., 2021). Early preventive interventions are usually implemented during the hospitalization in the NICU, with the aim of optimizing brain plasticity, creating and maintaining adequate neuronal connections. These interventions are designed to support the development of the very vulnerable premature infants and allowing them to develop stress regulation capacities (Kuhn et al., 2018; Symington and Pinelli, 2006; Westrup, 2016). More recently, various studies have been carried out on different types of multisensory enrichment during hospitalization to optimize the infant’s sensory experience and thus potentially improve development.

Early music interventions, particularly those centered on neonatal care, have been a subject of consistent inquiry, with research focusing on the immediate advantageous impacts of music listening and music therapy during the neonatal period.

Music listening involves exposure to recorded music, during which infants demonstrate receptiveness to a standard musical stimulus. A music therapy intervention consists of a systematic, interactive approach conducted by a qualified therapist, frequently utilizing live music to achieve designated therapeutic objectives.

In the NICU, music listening entails subjecting premature infants to standardized recorded music. Studies indicate that newborns are responsive to recorded auditory stimuli. Conversely, music therapy is a systematic and participatory intervention conducted by a qualified music therapist. It generally incorporates live music and is customized to address the individual requirements of each newborn. This interactive method fosters a dynamic and responsive treatment setting, potentially enhancing the efficacy of addressing the specific requirements of preterm newborns (Yue et al., 2021; Haslbeck, 2012).

A number of existing reviews and meta-analyses undertook the assessment of the impact stemming from early music and music therapy interventions on preterm infants within the NICU (Bieleninik et al., 2016; Haslbeck, 2012; Haslbeck et al., 2023; Palazzi et al., 2018; Van der Heijden et al., 2016; Yue et al., 2021). More specifically, evidence emerged on the impact of an early musical enrichment of the NICU environment on the structural and functional development of the premature infant’s brain (de Almeida et al., 2023; Loukas et al., 2022; de Almeida et al., 2020; Lordier et al., 2019a; Lordier et al., 2019b). However, the current body of research predominantly focuses on the immediate effects of music and music therapy during the neonatal period, creating a critical gap in our understanding of the enduring and sustained benefits or potential challenges that may arise over an extended period beyond the NICU stay.

While the aforementioned reviews offer valuable insights into the limited short-term effects of music and music therapy on preterm infants, particularly in areas such as heart rate, respiratory rates, feeding domains and parental anxiety, a more comprehensive understanding of their impact requires future investigations that extend their scope to encompass the long-term developmental trajectory of these infants, including their later emotional processing (Beltrán et al., 2022; Haslbeck et al., 2023). Addressing this research gap is essential for refining the efficacy of early music and music therapy interventions and informing holistic care strategies for preterm infants throughout their development.

The present study aims to comprehensively assess the impact of an early environmental enrichment through music during hospitalization on various facets of development, specifically focusing on temperament—including emotional abilities and attentional capacity, and effortful control abilities in a cohort of very VPT infants at 12-months and 24-months old, by comparing them to a control group of VPT infants and a group of full-term infants.

## 2 Method

### 2.1 Population

The original sample comprised 105 VPT (with a gestational age at birth of less than 32 weeks) and 48 infants born full-term (FT). These infants were part of a longitudinal study examining the impact of early music exposure during their stay in the NICU, as outlined in the studies by Lordier et al. (2019a,b) and de Almeida et al. (2023) on brain processing and neurobehavioral development. Recruitment took place at the University Hospital of Geneva, with written informed consent from the parents obtained before the participation of each newborn. The study adhered to the principles of the Declaration of Helsinki and received approval from the ethics committee at the University Hospital of Geneva. VPT were randomly allocated to either musical intervention group (55 VPT-music), or no-music intervention group (50 VPT-control).

The present study involved a comprehensive assessment of the motor, cognitive, language and emotional abilities of children aged 12 and 24 months, conducted within the follow-up unit of the University Hospital of Geneva. Three participants from the original cohort were excluded from the follow-up assessment. Exclusion criteria for all neonates included the presence of major brain lesions identified on early MRI, such as intraventricular hemorrhage grade III with or without apparent periventricular hemorrhagic infarction, or cystic periventricular leukomalacia (1 FT, 1 VPT-control) and genetic syndrome (1 VPT-music). Thirty-one infants did not participate to the follow-up due to parent refusal (5FT, 4 VPT-music, 6 VPT-control), move to another city/country (1FT, 6 VPT-music, 1 VPT-control), or because the parents were unreachable (5FT, 3 VPT-control). The neonatal characteristics of VPT infants who participated in the follow-up at 12 and 24 months compared with those who were lost to follow-up were not significantly different, in terms of sex, gestational age at birth, birth weight, and presence of bronchopulmonary dysplasia or sepsis. However, lower socioeconomic scores (meaning higher socioeconomic status; *p* = 0.026) of the children lost to follow-up (mean: 3.895 ± 1.969) was observed when compared to those who attended follow-up (mean: 5.215 ± 3.189).

**Figure 1:**
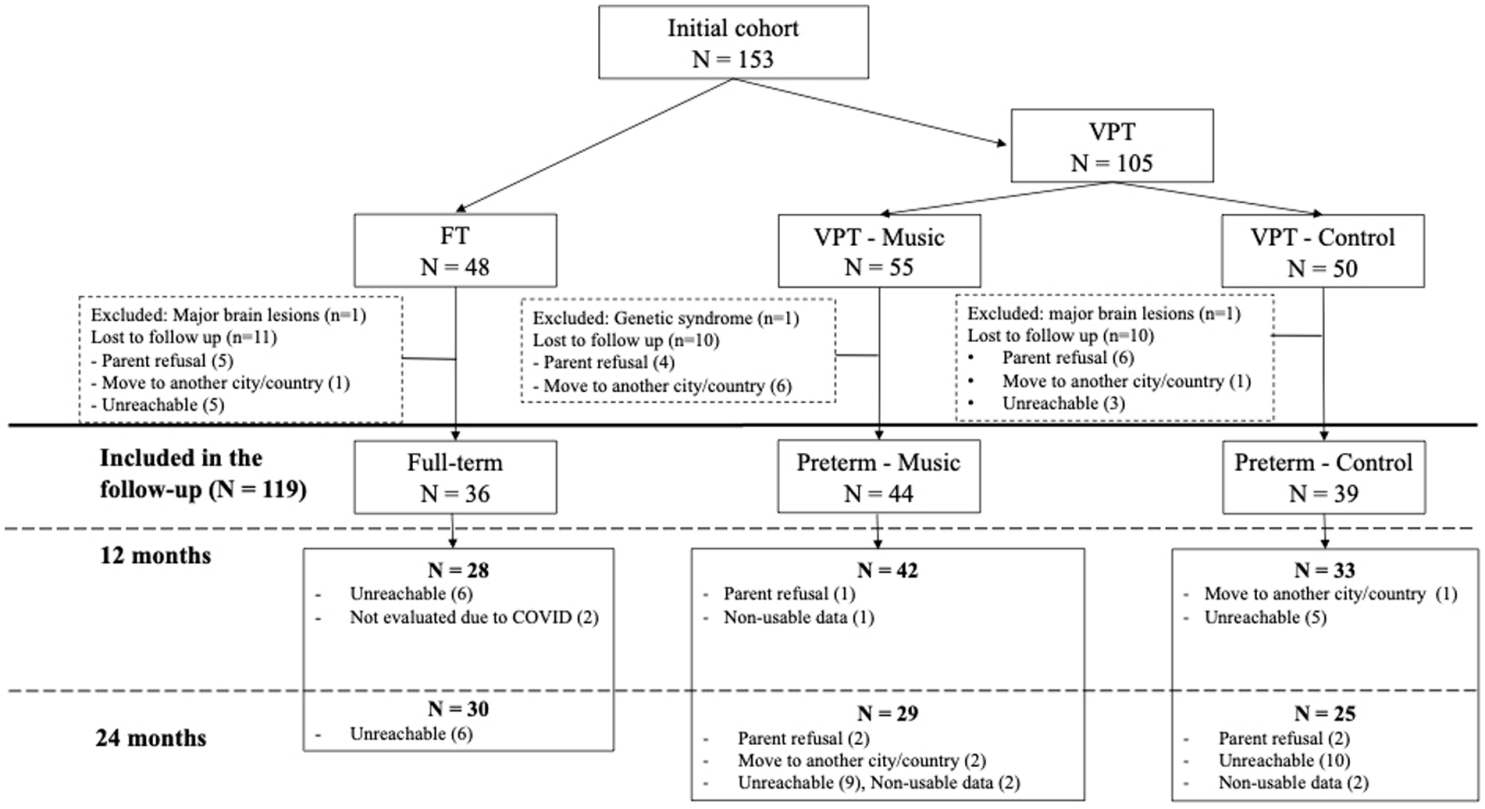
Flowchart illustrating the data collection process.

The final sample included 36 FT, 44 VPT-music (age range at birth, between 24 and 31.9 weeks of gestational age), and 39 VPT-control (age range at birth, between 24 and 32.57 weeks of gestational age, for details see Figure 1). At 12 months of corrected age, data were collected for 28 FT, 42 VPT-music and 33 VPT-control and at the corrected age of 24 months, information was gathered for 30 FT, 29 VPT-music and 25 VPT-control (for details see Figure 1).

### 2.2 Study design

#### 2.2.1 Intervention

Parents, caregivers, and providers of music intervention were blind to the group assignment.

The newborns in the control group were provided with headphones that were identical to those given to the neonates in the experimental group. However, it should be noted that these headphones were non-functional. Consequently, when the experimenter placed the headphones, it was impossible to ascertain if the infant was truly being exposed to the music or not. Using headphones, the VPT-music group listened to 8-min infant state adapted music extracts composed by Andreas Vollenweider,^1^ beginning at 33 weeks’ gestation and continuing until hospital discharge or term-equivalent age.

The infant was exposed to music in accordance with his biological rhythm and state of wakefulness: one composition was designed to assist the infant in awakening, another to sustain the infant in a state of tranquil awakening, and the final one to facilitate the infant’s descent into sleep.

To accommodate the infants’ states of wakefulness, the structures of the three pieces of music were different. The music designed to help the baby wake up started with a soothing background, with instruments gradually being introduced. Conversely, the music intended to help the baby fall asleep began with instruments, which were then gradually removed. For the music administered during the active state, instruments were evenly distributed throughout the piece. The auditory spectrum and the musical pieces can be found in our previously published paper (Lordier et al., 2019b).

The three pieces are stereophonic musical excerpts in B major, lasting 8 min each, composed by Andreas Wollenweider. The main foundation of the three pieces is a sound bed that features a multitude of harmonics originating from human voices. Musical elements such as harp, punji, and bells intermittently punctuate this vocal tapestry at various points within the stereophonic panorama.

The musical pieces utilize a tempo moderato and a steady rhythm that provide a consistent auditory experience. The harmonics from the human voices create a rich and layered sound bed, which serves as the primary backdrop of the composition. This vocal foundation is dynamically enriched with the occasional introduction of instrumental sounds. The first instruments that is gradually introduced in the musical piece is the harp. The delicate plucking of the harp strings adds a gentle quality to the music, enhancing the overall texture and providing melodic highlights. Secondly, the principal melody has been presented by the punji, a traditional wind instrument, introducing unique timbres and cultural flavors, contributing to the piece’s diversity. Six simple melodies played by the punji were repeated throughout the music extract. The mean pitch of the melodic lines played by the punjji is 319.2 Hz (range 320–360 Hz). Finally, the sporadic chimes of bells offer a percussive element that punctuates the soundscape, creating moments of clarity and accentuating the harmonic richness.

The three pieces present the same structure (Background + instrumental sounds), the same rhythm and tempo characteristics, they are in the same key, they only differ for the presentation of the instrumental sounds.

The music designed to help the baby wake up started with a soothing background, with instruments gradually being introduced. It presents a moderate crescendo in intensity in the central part. Conversely, the music intended to help the baby fall asleep began with instruments, which were then gradually removed and in the central part a moderate diminuendo gradually leads to the end of the piece. For the music administered during the active state, instruments were evenly distributed throughout the piece. The music extracts are presented in Lordier et al. (2019b) in the Supplementary material.

The infant’s readiness for music exposure and the appropriate track were determined using a neonatal behavioral assessment scale (Martinet et al., 2013).

The grid extensively describes the infant’s behavioral and physiological characteristics in the different sleep and awake states, guiding the nurses in observing the child’s behavior and physiology, including heart and respiratory rates, signs of discomfort, and self-regulation. All the nurses involved in the project were trained to the grid use. The music was not administered when the babies were in a deep sleep state or a crying state. After nutrition time, if the newborns were falling asleep, dedicated music for the sleep state was chosen. Before care procedures or nutrition times, when the baby was in an active sleep state and beginning to transition to the awake state, the third piece of music, designed for the sleep-to-awake state, was administered.

The intervention was exclusively executed in the infant’s crib. The sound level varied from 30 dBA (background) to 65 dBA (peak with the bells). More details about the music intervention can be found in Lordier et al. (2019).

VPT-music infants listened to music about 5 times per week and VPT-control infants had open headphones put on without music at the same frequency. Infants were presented with a music piece approximately 2 times per day, so a mean of 16 min per day.

The mean number of music experience was 46.76 (SD = 24.70) for the VPT-music group and the mean number of times having the open headphones was 43.5 (SD = 26.73) for the VPT-control group (*p* = 0.57). Headphones were specifically designed for the study and adapted to preterm infants’ head size (for a detailed description of the headphones including pictures, see Supplementary Figure S2 in de Almeida et al., 2023).

A cohort of FT infants was enrolled at the maternity unit of the hospital in their first days of life. They were subsequently contacted at 12 and 24 months for follow-up evaluations.

#### 2.2.2 Perinatal characteristics

Characteristics such as sex, gestational age, birth weight, presence of bronchopulmonary dysplasia or sepsis, were obtained from the birth record and are presented in Table 1.

**Table 1:** Population characteristics.

	VPT-control	VPT-music	Full-term	VPT-control vs. VPT-music
	*n* = 39	*n* = 44	*n* = 36	
	n*(%)* or mean*(SD)*	n*(%)* or mean*(SD)*	n*(%)* or mean*(SD)*	*p*-value^a^
Sex, number of females	22*(56%)*	20*(45%)*	21*(58%)*	ns 0.45
Gestational age at birth (weeks)	28.91*(2.2)*	28.78*(2.2)*	39.43*(1.1)*	ns 0.763
Birth weight (g)	1,147*(303)*	1,181*(419)*	3,261*(407)*	ns 0.888
Bronchopulmonary dysplasia	13*(33%)*	16(37%)	0	ns 0.773
Early and late onset sepsis	12*(31%)*	7*(16%)*	0	ns 0.108
Socioeconomic score^b^	5.33*(3.4)*	5.10*(3.2)*	4.14*(2.9)*	ns 0.791

a Pearson’s chi-square and Mann–Whitney U-tests were used for the comparison of the variables between the VPT-music, VPT-control groups.

b The socioeconomic status (SES) was calculated using the Largo et al. (1989) 12-point scale based on paternal occupation and maternal education (range from 2 – the highest SES – to 12 – the lowest SES).

The socioeconomic status (SES) of the families was calculated using the Largo et al. (1989) questionnaire. The SES was computed on a 12-point scale, considering both paternal occupation and maternal education, with scores ranging from 2 (indicating the highest SES) to 12 (representing the lowest SES).

#### 2.2.3 Cognitive, language, motor, and emotional assessment

Parents gave written consent for participation in the study and video recording of the assessment. Each child, in the presence of a caregiver, was assessed in a quiet room. Items were administered and scored by qualified psychologists and psychomotor therapist blinded to group assignment.

At 12 months of age (or corrected age for children born prematurely), cognitive, language and motor development were assessed using the Bayley scales of infant and toddler development third edition (Bayley-III) (Bayley, 2006). Four items from the laboratory temperament assessment battery (Lab-TAB), described in the paragraph on measures, were used to observe individual differences in emotional expression (fear, anger, joy), activity level (sustained attention) and regulatory aspects of behavior (Goldsmith and Rothbart, 1999). The child’s temperament was also assessed using the Infant Behavior Questionnaire (IBQ) (Rothbart, 1981).

At 24 months (or corrected age for children born prematurely), the children were also assessed using the Bayley-III and Lab-TAB. In addition, the ability to inhibit a motor response and delay gratification were assessed with 3 episodes of the Effortful Control Battery (ECB) (Kochanska et al., 2000). Parents also completed the short version of the Early Childhood Behavior Questionnaire (ECBQ) (Putnam and Rothbart, 2006).

### 2.3 Behavioral, emotional and neurodevelopmental measures

#### 2.3.1 Laboratory temperament assessment battery (Lab-TAB)

The 4 episodes of the Lab-TAB, a reliable and valid observation protocol, were the puppet game (assessing expressions of joy), the attractive toy placed behind a barrier (assessing expressions of anger), the unpredictable mechanical toy (to evaluate fear responses), and the blocks (to rate sustained attention). Lab-TAB coding involved facial, vocal, and bodily measures. For each episode, measures were derived from the LAB-TAB coding manual and coded on a scale from 0 to 3, with 3 indicating a stronger emotional response. Each emotion involves specific activation of different facial regions (Izard et al., 1983; Izard and Malatesta, 1987). Thus, coding facial intensity for anger and fear is based on the number of facial regions activated (e.g., a score of 3 is assigned if 3 specific emotion-related regions are activated). Coding details are in the Lab-TAB manual.

The puppet game assesses joy and involves the presentation of a scripted puppet show, lasting around 1 min, which tickles the child three times. Scoring was carried out for four equivalent time intervals (introduction, first tickle, second tickle and third tickle). A mean score of joy (smile intensity; positive vocalizations, for example babbling or squealing; touching puppets rate) was made. The intensity of negative motor activity was also coded (for example, continuous and brutal pushing away of the puppets was scored as 3). To assess anger, an attractive toy was presented to the child, then gently taken from his hands and placed behind a plexiglass barrier for 30 s. The task was repeated twice. Anger intensity (vocal, for example full intensity cry or scream was scored as 3; facial; and motor) was rated in 5-s intervals and averaged for each trial. An average anger score for each trial and for the entire task was calculated. Fear is assessed by a task with an unpredictable mechanical robot that moves toward the child, stops in front of him and makes grunts, then moves back. The episode lasted 15 s and was repeated twice. The mean of fear ratings (facial; vocal, for example mild vocalization that may be difficult to identify as hedonically negative was coded as 1; and bodily, for example visible and sustained tensing of the muscles, associated with decreased activity was coded as 2) was calculated for each trial. Positive motor actions (approaching the robot and trying to grasp it) were also rated for each trial. A mean score for fear and a mean score for positive motor action throughout the task were calculated. The Blocks episode measures the child’s sustained attention in free play with blocks for 3 min. Facial interest, gaze duration and object manipulation duration were scored in 10-s time intervals. All scores were averaged to calculate a sustained attention score for each minute and an average score for the 3 min.

#### 2.3.2 Effortful control battery

The 3 episodes of the Effortful Control Battery were Snack delay, Wrapped gift and Tower. The Snack Delay measures the child’s ability to delay gratification. The child must keep their hands on the table and not touch or eat the treat placed in front of them before the experimenter rings the bell. This task was presented 4 times, with delays of 10, 20, 30 and 15 s. In the middle of each trial, the experimenter lifted the bell but did not ring it. For each trial, an inhibition score was calculated on a scale of 1 to 9 (1 = the child ate a snack before the experimenter raised the bell; 7 = the child waited for the bell to ring). An extra point was awarded if the child kept his hands on the table at least half the time, and 2 points if he kept them there throughout the trial. An average inhibition score across the 4 trials was calculated. The Wrapped Gift also measures the child’s ability to delay gratification. The experimenter tells the child that he has a gift for him but has forgotten to wrap it. The child must wait with his back facing the experimenter and not look at the experimenter when he wraps the gift (60 s). The child thus obtains a score from 1 to 5 for turning to look at the gift wrapping: 1 = the child turns and continues to look; 3 = the child looks over his shoulder; 5 = the child does not look. Next, the wrapped gift is placed on the table and the experimenter leaves the room, telling the child not to touch the gift until he returns (180 s). The child obtains a score from 1 to 4: 1 = the child opens the gift; 4 = the child never touches the gift. The Tower assesses the ability to wait one’s turn by suppressing an impulsive motor response. The child must take turns building a tower with the experimenter. A first attempt is made to ensure that the child has understood the task. The episode comprised 2 trials, and an inhibition score was calculated for each trial. The total number of blocks placed (multiplied by 10) was divided by the number of blocks placed by the child. The maximum score (20) was obtained if the child waited his turn. A penalty of −5 points was applied if the child intentionally knocked over the tower. An average inhibition score combining the two trials was also calculated. Effortful Control examines the child’s ability to regulate attention and inhibit impulses, reflecting self-control and attentional focus. Negative Affectivity explores the extent to which the child displays negative emotions, such as anger, frustration, or fear. Surgency–Extraversion delves into traits related to positive anticipation, sociability, and approach behaviors, reflecting the child’s level of extroversion and enthusiasm in social interactions. These three subscales collectively contribute to a nuanced understanding of a child’s behavioral tendencies and emotional responses during early childhood.

#### 2.3.3 The infant behavior questionnaire (IBQ)

The questionnaire comprises 191 questions to which the parent answers from 1 (never) to 7 (always), and evaluated six domains of infant temperament, encompassing activity level, soothability, fear, distress in response to limitations, smiling and laughter, and the duration of orienting. Parents were asked to rate the frequency of temperament-related behaviors observed in their infants over the preceding week. Three subscales were utilized in the assessment, encompassing Surgency/Extraversion, Negative Affectivity, and Orienting Regulation. Surgency/Extraversion explores traits related to positive anticipation, sociability, and approach behaviors. Negative Affectivity delves into aspects associated with distress, fear, and frustration. Orienting Regulation focuses on the child’s ability to attend to and engage with environmental stimuli. These subscales collectively provide a comprehensive understanding of the infant’s temperament across different dimensions.

Not all the parents completed the extensive questionnaires, and the numbers of missing questionnaires was reported in Tables 2, 3. If a caregiver omitted an item, or if the caregiver checked the “Does not apply” response option for an item, the item receives no numerical score and is not factored into the scale score.

**Table 2:** Results of assessment at 12 months in the 3 groups.

	VPT-control	VPT-music	Full-term		
	mean*(SD)*	mean*(SD)*	mean*(SD)*	*F*	*p*
**Bayley scales**	*(n = 33)*	*(n = 42)*	*(n = 11)*		
Cognitive	102.73*(12.6)*	101.55*(10.8)*	110.00*(11.3)*	1.84	0.166
Language	92.00*(8.6)*a	93.43*(8.2)*a	99.73*(5.9)*b	3.51	**0.035**
Motor	93.33*(10.2)*a	93.26*(8.8)*a	105.73*(9.8)*b	7.12	**0.001**
**Lab-TAB**	*(n = 32)*	*(n = 41)*	*(n = 28)*		
**Puppet game**					
Joy	0.86*(0.54)*	0.99*(0.59)*	0.81*(0.49)*	0.059	0.594
Negative motor action	0.50*(0.41)*	0.38*(0.44)*	0.40*(0.54)*	0.722	0.597
**Toy behind barrier**					
Anger-Trial 1	0.81*(0.61)*	0.65*(0.61)*	0.61*(0.51)*	1,987	0.143
Anger-Trial 2	0.64*(0.46)*	0.76*(0.55)*	0.68*(0.50)*	0.639	0.530
Anger (mean)	0.79*(0.63)*	0.75*(0.58)*	0.64*(0.47)*	1,525	0.223
Distraction-Trial 1	1.09*(0.90)*	1.12*(0.83)*	0.76*(0.67)*	0.575	0.565
Distraction-Trial 2	1.42*(0.99)*	1.27*(0.92)*	1.03*(0.86)*	0.524	0.594
Distraction (mean)	1.26*(0.88)*	1.22*(0.76)*	0.91*(0.67)*	0.518	0.597
**Unpredictable toy**					
Fear-Trial 1	0.32*(0.27)*	0.26*(0.31)*	0.37*(0.28)*	1,844	0.164
Fear-Trial 2	0.30*(0.28)*a	0.22*(0.29)b*	0.54*(0.37)*c	10.09	**0.001**
Fear-Trial (mean)	0.31*(0.22)*a	0.25*(0.30)*a	0.44*(0.31)*b	5,162	**0.007**
Positive motor action-Trial 1	1.20*(0.94)*a	1.63*(0.97)b*	1.04*(0.88)c*	3,891	**0.024**
Positive motor action-Trial 2	1.31*(1.04)*a	1.62*(1.08)*b	0.98*(1.02)*c	3,235	**0.044**
Positive motor action (mean)	1.29(0.90)a	1.65*(0.99)*b	1.02*(0.88)*c	4,311	**0.016**
**Blocks**					
Sustained attention- Trial 1	2.58*(0.47)*	2.56*(0.40)*	2.41*(0.58)*	0.111	0.111
Sustained attention -Trial 2	2.21*(0.87)*	2.33*(0.62)	2.34*(0.58)*	0.591	0.591
Sustained attention-Trial 3	1.99*(0.90)*	2.20*(0.76)*	2.05*(0.69)*	0.555	0.555
Sustained attention (mean)	2.25*(0.67)*	2.36*(0.49)*	2.28*(0.53)*	0.463	0.463
**IBQ**	*(n = 17)*	*(n = 34)*	*(n = 29)*		
Extraversion	4.80*(0.72)*	5.11*(0.57)*	4.92(0.49)	0.098	0.143
Negative affectivity	3.96*(0.50)*	3.97*(0.56)*	4.24(0.44)	0.143	0.530
Orienting regulation	4.66*(0.58)*	4.85*(0.59)*	4.69(0.46)	0.373	0.223

Boldface entries indicate significant difference (*p* < 0.05); a,b,c: different letters significant difference at *p* < 0.05.

**Table 3:** Results of assessment at 24 months in the three groups.

	VPT-control	VPT-music	Full-term		
	mean*(SD)*	mean*(SD)*	mean*(SD)*	*F*	*p*
**Bayley scales**	*(n = 23)*	*(n = 31)*	*(n = 30)*		
Cognitive	98.26(9.24)	97.58(11.47)	103.0(10.05)	1,369	0.261
Language	90.52(9.43)	89.48(11.14)	94.53(10.43)	0.957	0.388
Motor	96.30(8.64)	96.45(10.81)	102.0(12.26)	1,958	0.148
**Lab-TAB**	*(n = 22)*	*(n = 27)*	*(n = 31)*		
**Puppet game**					
Joy	0.98*(0.68)*	1.02*(0.63)*	0.85*(0.54)*	1,209	0.304
Negative motor action	0.55*(0.53)*a	0.16*(0.27)*b	0.49*(0.59)*a	4,729	**0.012**
**Toy behind barrier**					
Anger-Trial 1	0.44*(0.33)*	0.68*(0.70)*	0.63*(0.40)*	0.879	0.420
Anger-Trial 2	0.41*(0.37)*	0.62*(0.72)*	0.71*(0.53)*	0.562	0.456
Anger (mean)	0.43*(0.31)*	0.63*(0.69)*	0.66*(0.43)*	0.921	0.403
Distraction-Trial 1	0.80*(0.84)*	0.80*(0.85)*	0.66*(0.96)*	0.144	0.866
Distraction-Trial 2	1.21*(1.0)*	1.10*(1.2)*	0.79*(0.98)*	0.681	0.509
Distraction (mean)	0.98*(0.91)*	0.96*(0.98)*	0.73*(0.88)*	0.414	0.663
**Unpredictable toy**					
Fear-Trial 1	0.81*(0.70)*	0.67*(0.56)*	0.61*(0.61)*	0.234	0.792
Fear-Trial 2	0.70*(0.62)*	0.73*(0.71)*	0.78*(0.71)*	0.316	0.730
Fear-Trial (mean)	0.83*(0.73)*	0.73*(0.63)*	0.69*(0.62)*	0.029	0.973
Mean Positive-Trial 1	0.86*(0.94)*	0.99*(1.2)*	0.96*(1.0)*	0.036	0.964
Mean Positive-Trial 2	0.77*(1.0)*	0.90*(1.1)*	0.83*(1.0)*	0.101	0.904
Mean Positive12	0.77*(0.88)*	0.91*(1.1)*	0.90*(0.91)*	0.035	0.966
**Blocks**					
Sustained attention- Trial 1	2.43(0.61)a	2.69(0.35)b	2.74(0.37)b	2,889	**0.062**
Sustained attention -Trial 2	2.42(0.63)	2.56(0.53)	2.48(0.70)	0.496	0.611
Sustained attention-Trial 3	2.35(0.70)	2.45(0.64)	2.20(0.82)	0.567	0.570
Sustained attention (mean)	2.38*(0.57)*	2.58*(0.38)*	2.47*(0.50)*	1,351	0.266
**Effortful control battery**					
**Snack delay**					
Inhibition-Trial 1	7.40*(2.7)*	7.82*(2.4)*	6.96*(2.7)*	0.648	0.526
Inhibition-Trial 2	7.24*(2.7)*	6.74*(3.0)*	7.15*(2.9)*	0.169	0.845
Inhibition-Trial 3	6.45*(1.2)*	6.29*(3.1)*	6.19*(1.9)*	0.164	0.849
Inhibition-Trial 4	7.20*(2.6)*	6.86*(2.8)*	7.28*(2.1)*	0.200	0.819
Inhibition-Trial (mean)	7.12*(2.3)*	6.96*(2.3)*	6.86*(2.2)*	0.040	0.960
**Tower**					
Inhibition-Trial 1	16.9*(2.3)*	15.6*(3.7)*	14.4*(4.0)*	1,849	0.165
Inhibition-Trial 2	15.6*(3.5)*	15.9*(3.6)*	15.0*(3.6)*	0.286	0.753
Tower inhibition (mean)	16.5*(2.4)*	15.7*(3.7)*	14.7*(3.5)*	1,059	0.352
**Wrapped gift**					
Peak and turn	1.90*(1.29)*	1.68*(1.03)*	1.83*(1.15)*	1,798	0.184
Touch	3.36*(1.14)*	3.11*(1.58)*	3.37*(1.03)*	0.284	0.753
**ECBQ**	*(n = 21)*	*(n = 22)*	*(n = 26)*		
Effortful Control	4.78*(0.57)*	4.75*(0.58)*	4.63*(0.49)*	0.878	0.421
Negative Affectivity	3.56*(0.66)*	3.31*(0.49)*	3.28*(0.40)*	1,070	0.349
Surgency–Extraversion	4.73*(0.62)*	4.73*(0.60)*	4.62*(0.47)*	0.223	0.801

Boldface entries indicate significant difference (*p* < 0.05); a,b,c: different letters significant difference at *p* < 0.05.

#### 2.3.4 Early childhood behavior questionnaire (ECBQ)

It includes 107 questions, with responses ranging from 1 (never) to 7 (always), and provides scores for: Activity Level, Attentional Focusing, Attentional Shifting, Cuddliness, Discomfort (amount of negative affect related to sensory qualities of light, sound, texture stimulations), Fear, Frustration, Low-intensity and High-intensity Pleasure, Impulsivity, Inhibitory Control, Motor Activation (i.e., repetitive small-motor movements), Perceptual Sensitivity, Positive Anticipation, Sadness, Shyness, Sociability, Soothability. Three subscales of the ECBQ offer insights into distinct aspects of a child’s temperament.

### 2.4 Data analysis

All statistical analyses were conducted using SPSS 27.0 (IBM SPSS Statistics, IBM Corporation).

To test for differences in demographic and perinatal data between groups, categorical variables were analyzed using chi-squared test, whereas continuous variables were compared using Mann–Whitney U-tests.

Analyses of covariance (ANCOVA) were used, with SES as a covariate (after controlling the insignificance of the relationship between SES and Group and the equality of slope of regression lines in the three groups). Analyses of covariance were performed for each dependent variable comparing the three groups (VPT-music vs. VPT-control vs. full-term) as between-subjects factor, with sex as additional between factor and SES as a covariate. The analyses were conducted separately for measures at 12 and 24 months.

Effect sizes and observed power for the overall ANCOVAs were reported, as well as post-hoc contrasts. The significant threshold was <=0.05 and the marginal threshold was <=0.07. In addition, we calculated the post-hoc achieved power with G*power 3.1, providing the given alpha level, effect size set as large, and actual sample size. The calculated achieved power is 0.97, which is considered adequate.

## 3 Results

No significant differences were found between the three groups (VPT-music, VPT-control and full-term) regarding the neonatal characteristics and the SES.

### 3.1 At 12 months

Table 2 presents the findings of the clinical assessment of children at 12 months corrected age.

No significant effect of the music intervention on Bayley-III scales, was observed at 12 months. For Language scores, there was a significant group effect (*F*(2,86) = 3.51, *p* = 0.035, *η*^2^_p_ = 0.082, observed power = 0.64), with higher scores for FT infants (*M* = 99.73, SD = 5.9) compared with the VPT-control group (*M* = 92.00, SD = 8.6) and the VPT-music group (*M* = 93.43, SD = 8.2). There was a significant effect of sex, *F*(1,86) = 6.70, *p* = 0.011, *η*^2^_p_ = 0.08, observed power = 0.73, with females obtaining significantly higher scores across all three groups (M_females = 95.91, SD = 9.4; M_males = 91.24, SD = 6.5). For Motor score, a similar pattern emerged, with a significant effect of group (*F*(2,86) = 7.12, *p* = 0.001, *η*^2^_p_ = 0.15, observed power = 0.92), with higher scores for FT infants (*M* = 105.73, SD = 9.8) compared with the VPT-control group (*M* = 93.33, SD = 10.2) and the VPT-music group (*M* = 93.26, SD = 8.8) and a significant effect of sex (*F*(1,86) = 4.197, *p* = 0.044, *η*^2^_p_ = 0.05, observed power = 0.53), with higher scores for females (M_females = 96.80, SD 10.8; M_males = 92.78, SD = 9.3). No significant effect of SES was observed, nor were there any significant interactions.

For the Lab-TAB assessment at 12 months, a significant effect of group emerged in the Unpredictable Toy episode for the variable Fear Trial 2 (*F*(2,94) = 10.09, *p* < 0.001, *η*^2^_p_ = 0.19, observed power = 0.98); and significant for Fear Trial Mean (*F*(2,100) = 5,162, *p* = 0.007, *η*^2^_p_ = 0.099, observed power = 0.82). A significant effect of group was observed also for the variables: Positive Motor Action for both Trial 1 (*F*(2,100) = 0.891, *p* = 0.024, *η*^2^_p_ = 0.08, observed power = 0.69), Trial 2 (*F*(2,94) = 3,235, *p* = 0.044, *η*^2^_p_ = 0.7, observed power = 0.60), and for Mean Positive Motor Action (*F*(2,100) = 4,311, *p* = 0.016, *η*^2^_p_ = 0.084, observed power = 0.74). Post-hoc analyses revealed that the VPT-music had significantly lower scores for Fear and higher scores for Positive Motor action compared to the VPT-control and full-term groups.

In addition, the Group x Sex interaction was found to be significant for Fear, Trial 2 (*F*(2,86) = 3.846, *p* = 0.025, *η*^2^ = 0.076, observed power = 0.69) and Fear Mean (*F*(2,86) = 3.846, *p* = 0.025, *η*^2^_p_ = 0.076, observed power = 0.69), as well as for Positive Motor Action, Trial 2 (*F*(2,86) = 3.846, *p* = 0.025, *η*^2^_p_ = 0.076, observed power = 0.69) and Positive Motor Action Mean value (*F*(2,86) = 3.846, *p* = 0.025, *η*^2^ = 0.076, observed power = 0.69). For Fear Mean, the full-term subgroup males showed higher scores than females (the difference was significant at the t-test, *t*(27) = 2.15, *p* = 0.018), while the sex differences were not significant in the VPT-music and VPT-control groups. For Positive Motor Action Mean, the VPT-music subgroup males had higher scores than females (the difference was significant at the t-test, *t*(38) = 2.42, *p* = 0.021). No effect of SES was observed.

Moreover, significant effects of sex across the three groups emerged for Sustained Attention, at Trial 3 (*F*(1,97) = 6.455, *p* = 0.013, *η*^2^_p_ = 0.066, observed power = 0.71). Scores were significantly higher for males (Trial 3: M_males = 2.32, SD_males = 0.68 vs. M_females = 1.91, SD_females = 0.82; Mean value: M_males = 2.41, SD_males = 0.53 vs. M_females = 2.22, SD_females = 0.57).

Finally, significant effects for Sustained Attention Mean value (*F*(1,103) = 4.28, *p* = 0.041, *η*^2^_p_ = 0.042, observed power = 0.82). For the three dimensions of IBQ, no significant effect was observed.

### 3.2 At 24 months

Table 3 presents the findings from the clinical assessment of preterm-born children at 24 months corrected age.

No significant effect of the music intervention on Bayley-III scales was observed at 24 months. Ancova results revealed a significant effect of sex for Cognitive scores (*F*(1,83) = 4.432, *p* = 0.039, *η*^2^*_p_* = 0.054, observed power = 0.55), with higher scores for females in all three groups (M_females = 101.83, SD = 10.65; M_males = 97.67, SD = 10.2). Additionally, a significant effect of SES was observed (*F*(1,83) = 4.166, *p* = 0.045, *η*^2^*_p_* = 0.051, observed power = 0.52). For Language score, a similar pattern emerged, with a significant effect of sex (*F*(1,83) = 5.511, *p* = 0.021, *η*^2^*_p_* = 0.07, observed power = 0.64), again with higher scores for females (M_females = 93.98, SD = 9.30; M_males = 89.28, SD = 11.28), Additionally, also a significant effect of SES was observed (*F*(1,83) = 5.673, *p* = 0.020, *η*^2^*_p_* = 0.024, observed power = 0.21). Correlations indicated that as SES scores decrease, scores on both Bayley scales increase (cognitive: *r* = −0.21, *p* = 0.05; language: *r* = −0.23, *p* = 0.03), suggesting that higher levels of family SES are associated with higher cognitive and language scores. No significant effects were found for the Motor Scores.

At 24 months, for the 4 episodes of the Lab-TAB and the 3 tasks of the Effortful Control Battery, the only significant effect of group emerged for Negative Motor Actions (*F*(2,83) = 4.729, *p* = 0.012, *η*^2^*_p_* = 0.11, observed power = 0.78) of the Puppet game. Post-hoc analyses showed that VPT-music had significantly lower scores compared to both VPT-control and full-term groups (*p* < 0.05). No other significant effects emerged.

No significant group effect was found for the three dimensions of ECBQ.

## 4 Discussion

This study examines how early environmental enrichment through music during hospitalization affects infants’ temperament - including emotional abilities and attentional capacity, effortful control and neurodevelopmental outcomes in VPT infants at 12 and 24 months corrected age.

Significant effects were observed on few aspects of the infant’s emotional abilities at 12 months, with medium-large effects sizes. The VPT-music had significantly lower scores for Fear and higher scores for Positive Motor action compared to the VPT-control and full-term groups. Note that the coding of the variable Positive Motor action encompassed all movements involving pointing, reaching or grabbing the mechanical toy during the fear test. Infants who were exposed to music attempted to grasp the object, thus performing a proximity action in relation to the task, as opposed to a freezing response, typical of a fear reaction. The observed diminished fear response accompanied by an increased positive motor action to an apparently frightening object in the VPT-music group should be interpreted considering previously reported results in the literature and prior findings related to the brain effects of music listening in the NICU. Langerock et al. (2013) have previously shown that preterm infants present decreased fear levels at 12-months in comparison to full-term newborns when assessed using Lab-TAB battery. This finding at 12 months of age was interpreted as a developmental delay in preterm infants regarding preterm infants’ fear response at an early stage. However, decreased fear response to a mechanical toy could also be interpreted as a better emotion regulation. It should be noted that our study shows no significant difference in fear responses assessed by Lab-TAB at 12- and 24-months between VPT- control and full-term infants.

Interestingly, our previous pilot study suggested the potential long-term benefits of early music exposure on preterm infants’ temperament at 12 and 24 months of age (Lejeune et al., 2019). Results showed that the scores of VPT were different from those of full-term children for fear reactivity at 12 months and for anger reactivity at 24 months. These significant differences were less important between the VPT group receiving a music intervention and the full-term group than between the VPT group without music intervention and the full-term group. These results, although significant, were obtained with a small sample size.

Of notice, the decreased fear response that we find in the music group at 12 months was no longer significantly different from the other groups at 24 months, which is consistent with previous findings from our pilot study showing no differences between groups regarding fear reactivity by 24 months (Lejeune et al., 2019). Our results suggest thus an effect of the music intervention specifically in early emotional regulation maturation at 12 months, namely in fear responsivity, but no longer evident at 24 months, when also no differences are observed between VTP-control infants and full-term newborns.

Note that no significant differences were found in both IBQ at 12 months and ECBQ at 24 months questionnaires regarding the fear reaction between the three groups.

This response is likely a transient and subtle effect, influenced by other factors such as maternal sensitivity, which we plan to control for in future studies (Gartstein et al., 2018).

Music’s impact on emotional regulation has been thoroughly studied in different educational and therapeutic environments. Music has demonstrated significant impacts on mood control, stress alleviation, and emotional communication (Juslin and Västfjäll, 2008). Music has the potential to adjust arousal levels and elicit various emotions, making it an effective therapeutic tool, improving emotional well-being and resilience, among other things (Fancourt et al., 2014).

Neuroimaging studies have shown that children and young adults born preterm frequently show a reduction in the volume of important brain structures, such as the amygdala and hippocampus. In particular, FT infants were shown to present larger amygdala volumes in comparison to VPT at term-equivalent age (Cismaru et al., 2016; de Almeida et al., 2020). This same early music intervention, delivered during the NICU stay, was shown to significantly increase amygdala volumes in VPT infants exposed to music, compared to those not exposed to music, at term-equivalent age. In fact, amygdala volumes of VPT infants exposed to the music intervention were similar to those of full term newborns (de Almeida et al., 2020). Furthermore, VPT infants exposed to the music intervention also presented an increased maturation of the uncinate fasciculus at term-equivalent age, in comparison to the VPT-control group (de Almeida et al., 2020), as well as a significantly longitudinal increased maturation of the cortical insulo-orbito-temporopolar complex (de Almeida et al., 2023). The uncinate fasciculus is an associative white matter tract connecting parts of the limbic system in the temporal lobe, comprising the hippocampus and amygdala, with frontal regions, such as the orbito-frontal cortex. The orbito-temporopolar cortical complex corresponds to a cortical structural that is connected by the uncinate fasciculus. All these structures are involved in processing of affective stimuli (Kringelbach, 2005), evaluation of emotional association (Wildgruber et al., 2005) and top-down modulation of behavior (Ghashghaei and Barbas, 2002). These regions mentioned are thus essential parts of the fear conditioning circuitry in the brain, with key functions in processing and controlling emotions, especially fear and anxiety. Here we demonstrated that VPT infants exposed to music showed decreased fear behaviors and displayed more positive motor action. Early exposure to music may also enhance the development of adaptive fear responses, reflecting a more advanced emotional regulatory ability. However, further studies correlating the brain structure with clinical behavioral assessments are needed to better understand this relationship.

At 24 months, VPT-music group had lower scores for negative motor actions in the joy task, compared to both VPT-control and FT groups. In this episode, negative motor actions indicate that the child is unwilling to participate in joyful, playful tasks, suggesting a reduced level of engagement in shared playful activities. Children usually show happiness by actively engaging and interacting during these activities. The VPT-music group showed less negative motor behaviors, which can be interpreted as a greater willingness to engage and interact in activities aimed at evoking joy and play, showing less reluctance or refusal behaviors.

Exposure to music at a young age may therefore have a positive impact on a child’s inclination to participate in enjoyable and playful activities (Croom, 2015). The results suggest that exposure to music at a young age may improve a child’s emotional responsiveness and participation in pleasurable activities.

Lastly, a potentially interesting finding emerged regarding the tendency toward higher scores for sustained attention at Minute 1 at 24 months of age among the VPT group who received a music intervention. The importance of this finding highlights the potential benefits of early music intervention to mitigate attention-related challenges in VPT-born children and adolescents. Extensive research has demonstrated that VPT-born children face a significantly higher risk of attention-deficit disorder compared to their full-term counterparts (Treyvaud et al., 2014). Given the importance of sustained attention in academic, social, and daily functioning, interventions aimed at enhancing attentional abilities hold considerable promise for improving outcomes in this population. The early exposure to music, as observed in our study, appears to play a role in sustaining attention levels in preterm infants. However, it is crucial to interpret our findings with caution, because although promising, the contrast are not significant, only a tendency toward better scores are highlighted and limited to the first minute of the task.

It is worth to note that at 12 months, no significant differences were found between the two VPT groups in terms of cognitive, language and motor scores as assessed by the Bayley-III scales and at 24 months no significant differences were found between all groups which speaks to our overall well developing preterm groups.

Although early music and vocal interventions have demonstrated beneficial effects in cognitive and affective brain regions right after the interventions (Kostilainen et al., 2021; de Almeida et al., 2020; Lordier et al., 2019; Webb et al., 2015), the persistence of these effects and their influence on infants’ neurodevelopment remains a complex issue to evaluate. The present results are in line of most of the studies investigating the long-term impact on infant’s cognitive, linguistic and motor development, which found no relevant effect (Kostilainen et al., 2023; Haslbeck et al., 2021; Lejeune et al., 2019). It is also crucial to acknowledge that the Bayley-III has been subjected to criticism (Månsson et al., 2021), particularly for its sensitivity to linguistic and vocal affective nuances. This sensitivity would be enhanced by a careful acoustical and “musical” evaluation, which would enable the disentanglement of the emotional prosody perception and production abilities in the preverbal and early verbal period.

SES was a significant predictor of cognitive and language scores at 24 months, with a more favorable SES correlating with higher Bayley-III scores in these domains. These findings are consistent with previous studies, identifying SES as a significant prognostic factor of premature infants’ cognitive outcome during infancy and early childhood (Gui et al., 2019; Linsell et al., 2015; Pittet-Metrailler et al., 2019). Interestingly, females presented significantly higher cognitive and language Bayley-III scores at 24 months across groups. Similar findings have been reported in literature, showing that male preterm infants are at higher risk of cognitive impairment in comparison to females (Hintz et al., 2006; Skiöld et al., 2014; Wood et al., 2005).

Our study suggests thus that early music listening in the NICU may impact later emotional regulation and responsivity, as well attention capacities in children. Further research with larger sample sizes and extended follow-up periods is warranted to validate and extend our preliminary findings.

## 5 Limitations

One of the major limitations of the present study is the small sample size, partly due to the halt in recruitment during the COVID-19 pandemic, and partly due to high rate of attrition, with many parents finding it difficult to return for follow-up assessments and to complete questionnaires.

Surprisingly, the socio-economic status (SES) of the parents of the children lost to follow-up at 12 and 24 months was higher than that of the children who took part in the follow-up. This could be attributed to the fact that, in high SES families, both parents often work, which may limit their availability and time to participate in additional studies. In addition, these families often move because of professional obligations, which makes it more difficult to maintain contact with the clinic and results in a higher drop-out rate.

Another major limitation is that measures of the recruited children’s daily musical experience at home were not collected. It is known that practicing a musical experience in a home-based environment can impact on infant’s development, with important effects, between others, on language acquisition (Franco et al., 2024). In the present intervention, no sustained musical activities with family members were supported and supplemented by musical activities at home during the infant’s first months. In fact, during the music listening experience the babies were laying in their bed. The music was played mostly in a moment when the parents could not be present, with the idea of providing a sensory stimulus to the baby, when no other interaction was being provided. Moreover, we have no information on whether the control group was exposed to music apart from the study, either during their hospital stay or at home. Therefore, we cannot exclude the possibility that the results were influenced by parents ‘or carers’ non-monitored music exposure. This limitation emphasizes the need for future research to pay meticulous attention to the monitoring and regulation of auditory stimulation outside the study design periods.

Another limitation is the lack of highly sensitive tools for behavioral assessment of emotional regulation in early infancy. Future research should focus on these limitations to better understand the long-term effects of a neonatal music intervention. Additionally, the present intervention was based on a recorded stimulus. The administration of a live and directed musical intervention could offer to the preterm newborns an optimal bioecological niche for developing emotional relationship with carers (Browne, 2017).

Moreover, with the present experimental design, we cannot determine if the overmentioned effects were directly related to the music selection offered instead of culturally relevant or family preferred music, as there has been no comparison between familiar and unfamiliar musical pieces. However, based on our previous findings, we can assess that following the continuous exposition to the same musical pieces during the hospital stay, a memory and a degree of familiarity to the musical stimulus is evidenced at a brain level (Loukas et al., 2022).

To conclude, we can hypothesize that during pregnancy a familiarity and a preference for the songs of kin is built (Loewy, 2015), but we can also add that the exposition to new musical pieces impacts on the resting state of preterm infants exposed to music.

Finally, in general, evaluating the long-term clinical effects of early intervention studies using music as an enriching stimulus presents major challenges. These challenges arise for three primary reasons. Firstly, the study population is frequently limited and focused on short-term outcomes, which can hinder a comprehensive understanding of long-term effects. Secondly, the post-hospitalization phase is influenced by numerous variables, particularly those tied to the caregiver-child relationship and the socio-economic environment, which impact the musical environment at home and the ability to pursue subsequent musical experiences.

Moreover, the influence of post-hospitalization factors is crucial for infant’s development. We hypothesize that integrating families into music interventions might help to achieving lasting effects beyond the initial neonatal period. Thirdly, a crucial aspect that remains uncertain is how long the structural and functional changes in the infant brain associated with early music intervention, persist.

The dynamic nature of brain development underscores the complexity of this question. While early music and voice interventions have shown promise in inducing positive changes in both cognitive and affective brain areas (de Almeida et al., 2020; Kostilainen et al., 2021; Lordier et al., 2019b; Webb et al., 2015), the stability of these effects remains challenging to ascertain. Continued research and longitudinal studies are essential to unravel the persistence of these positive modifications at a brain level over time.

### 5.1 Suggestions for future practice and research implications

To summarize, the overmentioned limitations should be addressed in future research and practice. First, unmonitored music exposure at home or in the hospital may affect research results, thus future studies should monitor and regulate auditory stimulation beyond the study times. Sustained musical activities at home, possibly with family members, may enhance linguistic and emotional development in infants.

Future study should use bigger sample numbers to overcome recruitment and attrition, especially during follow-up evaluations, and data on the infant’s daily musical experiences at home might help explain how home-based exposure affects development. The lack of sensitive measures to assess emotional regulation in early infancy shows a gap in our knowledge of newborn music therapies’ long-term consequences, which should be studied in future investigations. Finally, longitudinal studies are needed to determine how long early music exposure’s positive effects on brain development last.

## 6 Conclusion

The robustness and generalizability of the long-term effects of early music intervention for preterm newborns in the NICU remains challenging. Promising effects on children’s emotional development have been highlighted in the present study, but larger sample sized studies are needed to evaluate the clinical impact of a music intervention, that has demonstrated brain changes at term equivalent age (Lordier, 2019; Haslbeck et al., 2020; Kostilainen et al., 2021; de Almeida et al., 2023).

Understanding the long-term impact of early music interventions on preterm infant’s clinical outcomes is essential for adapting effective strategies that support positive actions and guide developmental music interventions throughout the child’s growth trajectory.

## Data Availability

The raw data supporting the conclusions of this article will be made available by the authors, without undue reservation.

## Footnotes

^1^ http://vollenweider.com/en

## References

[ref1] Arpi E. Ferrari F. (2013). Preterm birth and behaviour problems in infants and preschool-age children: a review of the recent literature. Dev. Med. Child Neurol. 55, 788–796. doi: 10.1111/dmcn.12142, PMID: 23521214

[ref2] Bayley N. (2006). Bayley scales of infant and toddler development. Development 3rd Edition: Screening Test Manual. San Antonio, TX: Harcourt Assessment, Inc.

[ref3] Beltrán M. I. Dudink J. de Jong T. M. Benders M. J. van den Hoogen A. (2022). Sensory-based interventions in the NICU: systematic review of effects on preterm brain development. Pediatr. Res. 92, 47–60. doi: 10.1038/s41390-021-01718-w, PMID: 34508227

[ref4] Berking M. Wupperman P. (2012). Emotion regulation and mental health: recent findings, current challenges, and future directions. Curr. Opin. Psychiatry 25, 128–134. doi: 10.1097/YCO.0b013e3283503669 22262030

[ref5] Bhutta A. T. Cleves M. A. Casey P. H. Cradock M. M. Anand K. J. (2002). Cognitive and behavioral outcomes of school-aged children who were born preterm: a meta-analysis. JAMA 288, 728–737. doi: 10.1001/jama.288.6.728, PMID: 12169077

[ref6] Bieleninik Ł. Ghetti C. Gold C. (2016). Music therapy for preterm infants and their parents: a meta-analysis. Pediatrics 138. doi: 10.1542/peds.2016-0971, PMID: 27561729

[ref7] Browne J. V. (2017). “Recorded maternal voice, recorded music, or live intervention: a bioecological perspective” in Early vocal contact and preterm infant brain development (Springer US: Springer), 183–201.

[ref8] Cassiano R. G. Provenzi L. Linhares M. B. M. Gaspardo C. M. Montirosso R. (2020). Does preterm birth affect child temperament? A meta-analytic study. Infant Behav. Dev. 58:101417. doi: 10.1016/j.infbeh.2019.101417, PMID: 31927307

[ref9] Chen F. Bajwa N. M. Rimensberger P. C. Posfay-Barbe K. M. Pfister R. E. (2016). Thirteen-year mortality and morbidity in preterm infants in Switzerland. Arch Dis Child Fetal Neonatal Ed 101, F377–F383. doi: 10.1136/archdischild-2015-308579, PMID: 27059074

[ref10] Cheong J. L. Burnett A. C. Treyvaud K. Spittle A. J. (2020). Early environment and long-term outcomes of preterm infants. J. Neural Transm. 127, 1–8. doi: 10.1007/s00702-019-02121-w 31863172

[ref11] Cisler J. M. Olatunji B. O. (2012). Emotion regulation and anxiety disorders. Curr. Psychiatry Rep. 14, 182–187. doi: 10.1007/s11920-012-0262-2, PMID: 22392595 PMC3596813

[ref12] Cismaru A. L. Gui L. Vasung L. Lejeune F. Barisnikov K. Truttmann A.. (2016). Altered amygdala development and fear processing in prematurely born infants. Front. Neuroanat. 10:55. doi: 10.3389/fnana.2016.00055 27242451 PMC4870280

[ref13] Clark C. A. Woodward L. J. Horwood L. J. Moor S. (2008). Development of emotional and behavioral regulation in children born extremely preterm and very preterm: biological and social influences. Child Dev. 79, 1444–1462. doi: 10.1111/j.1467-8624.2008.01198.x, PMID: 18826535

[ref14] Croom A. M. (2015). Music practice and participation for psychological well-being: a review of how music influences positive emotion, engagement, relationships, meaning, and accomplishment. Music. Sci. 19, 44–64. doi: 10.1177/1029864914561709

[ref15] de Almeida J. S. Baud O. Fau S. Barcos-Munoz F. Courvoisier S. Lordier L.. (2023). Music impacts brain cortical microstructural maturation in very preterm infants: a longitudinal diffusion MR imaging study. Dev. Cogn. Neurosci. 61:101254. doi: 10.1016/j.dcn.2023.101254, PMID: 37182337 PMC10200857

[ref16] de Almeida J. S. Lordier L. Zollinger B. Kunz N. Bastiani M. Gui L.. (2020). Music enhances structural maturation of emotional processing neural pathways in very preterm infants. NeuroImage 207:116391. doi: 10.1016/j.neuroimage.2019.116391, PMID: 31765804

[ref17] Fancourt D. Ockelford A. Belai A. (2014). The psychoneuroimmunological effects of music: a systematic review and a new model. Brain Behav. Immun. 36, 15–26. doi: 10.1016/j.bbi.2013.10.014, PMID: 24157429

[ref18] Forcada-Guex M. Borghini A. Pierrehumbert B. Ansermet F. Muller-Nix C. (2011). Prematurity, maternal posttraumatic stress and consequences on the mother–infant relationship. Early Hum. Dev. 87, 21–26. doi: 10.1016/j.earlhumdev.2010.09.006, PMID: 20951514

[ref19] Franco F. Chifa M. Politimou N. (2024). Home musical activities boost premature infants’ language development. Children 11:542. doi: 10.3390/children11050542, PMID: 38790537 PMC11120229

[ref20] Gardner F. Johnson A. Yudkin P. Bowler U. Hockley C. Mutch L.. (2004). Extremely low gestational age steering group. Behavioral and emotional adjustment of teenagers in mainstream school who were born before 29 weeks' gestation. Pediatrics 114, 676–682. doi: 10.1542/peds.2003-0763-L, PMID: 15342838

[ref21] Gartstein M. A. Hancock G. R. Iverson S. L. (2018). Positive affectivity and fear trajectories in infancy: contributions of mother–child interaction factors. Child Dev. 89, 1519–1534. doi: 10.1111/cdev.12843, PMID: 28542794 PMC5701886

[ref22] Ghashghaei H. T. Barbas H. (2002). Pathways for emotion: interactions of prefrontal and anterior temporal pathways in the amygdala of the rhesus monkey. Neuroscience 115, 1261–1279. doi: 10.1016/S0306-4522(02)00446-3, PMID: 12453496

[ref23] Goldsmith H. Rothbart M. (1993). The laboratory temperament assessment battery (LAB-TAB). Madison, WI: University of Wisconsin.

[ref24] Goldsmith H. Rothbart M. (1999). Laboratory temperament assessment battery (lab-TAB): Locomotor version 3.1. Available from H. Hill Goldsmith Ph.D. Personality Development Laboratory, Department of Psychology. Eugene, OR: University of Oregon.

[ref25] Gui L. Loukas S. Lazeyras F. Hüppi P. Meskaldji D. E. Tolsa C. B. (2019). Longitudinal study of neonatal brain tissue volumes in preterm infants and their ability to predict neurodevelopmental outcome. Neuroimage 185, 728–741. doi: 10.1016/j.neuroimage.2018.06.034, PMID: 29908311

[ref26] Haslbeck F. B. (2012). Music therapy for premature infants and their parents: an integrative review. Nord. J. Music. Ther. 21, 203–226. doi: 10.1080/08098131.2011.648653

[ref27] Haslbeck F. B. Bucher H. U. Bassler D. Hagmann C. Natalucci G. (2021). Creative music therapy and neurodevelopmental outcomes in pre-term infants at 2 years: a randomized controlled pilot trial. Front. Pediatr. 9:660393. doi: 10.3389/fped.2021.660393, PMID: 34222141 PMC8249730

[ref28] Haslbeck F. B. Jakab A. Held U. Bassler D. Bucher H. U. Hagmann C. (2020). Creative music therapy to promote brain function and brain structure in preterm infants: a randomized controlled pilot study. Neuroimage 25:102171. doi: 10.1016/j.nicl.2020.102171, PMID: 31972397 PMC6974781

[ref29] Haslbeck F. B. Mueller K. Karen T. Loewy J. Meerpohl J. J. Bassler D. (2023). Musical and vocal interventions to improve neurodevelopmental outcomes for preterm infants. Cochrane Database Syst. Rev. doi: 10.1002/14651858.CD013472.pub2 PMC10483930 37675934

[ref30] Healy E. Reichenberg A. Nam K. W. Allin M. P. Walshe M. Rifkin L.. (2013). Preterm birth and adolescent social functioning–alterations in emotion-processing brain areas. J. Pediatr. 163, 1596–1604. doi: 10.1016/j.jpeds.2013.08.011, PMID: 24070828

[ref31] Hintz S. R. Kendrick D. E. Vohr B. R. Poole W. K. Higgins R. D. Network N. N. R. (2006). Gender differences in neurodevelopmental outcomes among extremely preterm, extremely-low-birthweight infants. Acta Paediatr. 95, 1239–1248. doi: 10.1080/08035250600599727, PMID: 16982497

[ref32] Inder T. E. Volpe J. J. Anderson P. J. (2023). Defining the neurologic consequences of preterm birth. N. Engl. J. Med. 389, 441–453. doi: 10.1056/NEJMra2303347, PMID: 37530825

[ref33] Izard C. E. Dougherty L. M. Hembree E. A. (1983). A system for identifying affect expressions by holistic judgments (AFFEX). Newark: Instructional Resources Center, University of Delaware.

[ref34] Izard C. E. Malatesta C. Z. (1987). Perspectives on emotional development I: differential emotions theory of early emotional development. The first draft of this paper was based on an invited address to the Eastern Psychological Association, Apr 1, 1983.

[ref35] Johnson S. Marlow N. (2011). Preterm birth and childhood psychiatric disorders. Pediatr. Res. 69, 11R–18R. doi: 10.1203/PDR.0b013e318212faa0 21289534

[ref36] Johnson S. Marlow N. (2017). Early and long-term outcome of infants born extremely preterm. Arch. Dis. Child. 102, 97–102. doi: 10.1136/archdischild-2015-309581 27512082

[ref37] Joseph H. M. Lorenzo N. E. Fisher N. Novick D. R. Gibson C. Rothenberger S. D.. (2023). Research review: a systematic review and meta-analysis of infant and toddler temperament as predictors of childhood attention-deficit/hyperactivity disorder. J. Child Psychol. Psychiatry 64, 715–735. doi: 10.1111/jcpp.13753, PMID: 36599815 PMC10404471

[ref38] Juslin P. N. Västfjäll D. (2008). Emotional responses to music: the need to consider underlying mechanisms. Behav. Brain Sci. 31, 559–575. doi: 10.1017/S0140525X08005293, PMID: 18826699

[ref39] Klein V. C. Rocha L. C. Martinez F. E. Putnam S. P. Linhares M. B. M. (2013). Temperament and behavior problems in toddlers born preterm and very low birth weight. Span. J. Psychol. 16:E18. doi: 10.1017/sjp.2013.30, PMID: 23866211

[ref40] Kochanska G. Murray K. T. Harlan E. T. (2000). Effortful control in early childhood: continuity and change, antecedents, and implications for social development. Dev. Psychol. 36, 220–232. doi: 10.1037/0012-1649.36.2.220, PMID: 10749079

[ref41] Kostilainen K. Hugoson P. Haavisto A. Partanen E. Mikkola K. Huotilainen M.. (2023). No impact of parental singing during the neonatal period on cognition in preterm-born children at 2–3 years. Acta Paediatr. 112, 1471–1477. doi: 10.1111/apa.16788, PMID: 37026177

[ref42] Kostilainen K. Partanen E. Mikkola K. Wikström V. Pakarinen S. Fellman V.. (2021). Repeated parental singing during kangaroo care improved neural processing of speech sound changes in preterm infants at term age. Front. Neurosci. 15:686027. doi: 10.3389/fnins.2021.686027, PMID: 34539329 PMC8446605

[ref43] Kringelbach M. L. (2005). The human orbitofrontal cortex: linking reward to hedonic experience. Nat. Rev. Neurosci. 6, 691–702. doi: 10.1038/nrn1747, PMID: 16136173

[ref44] Kuhn P. Sizun J. Casper C. the GREEN study group from the French Neonatal Society (2018). Recommendations on the environment for hospitalised newborn infants from the French neonatal society: rationale, methods and first recommendation on neonatal intensive care unit design. Acta Paediatr. 107, 1860–1866. doi: 10.1111/apa.14501, PMID: 30025190

[ref45] Lammertink F. Vinkers C. H. Tataranno M. L. Benders M. J. (2021). Premature birth and developmental programming: mechanisms of resilience and vulnerability. Front. Psych. 11:531571. doi: 10.3389/fpsyt.2020.531571, PMID: 33488409 PMC7820177

[ref46] Langerock N. De Jonge L. V. H. Graz M. B. Hüppi P. Tolsa C. B. Barisnikov K. (2013). Emotional reactivity at 12 months in very preterm infants born at< 29 weeks of gestation. Infant Behav. Dev. 36, 289–297. doi: 10.1016/j.infbeh.2013.02.006, PMID: 23545077

[ref47] Largo R. Pfister D. Molinari L. Kundu S. Lipp A. Due G. (1989). Significance of prenatal, perinatal and postnatal factors in the development of AGA preterm infants at five to seven years. Dev. Med. Child Neurol. 31, 440–456. doi: 10.1111/j.1469-8749.1989.tb04022.x, PMID: 2680687

[ref48] Lejeune F. Borradori Tolsa C. Bickle Graz M. Hüppi P. S. Barisnikov K. (2015). Emotion, attention, and effortful control in 24-month-old very preterm and full-term children. Annee Psychol. 115, 241–264.

[ref49] Lejeune F. Lordier L. Pittet M. P. Schoenhals L. Grandjean D. Hüppi P. S.. (2019). Effects of an early postnatal music intervention on cognitive and emotional development in preterm children at 12 and 24 months: preliminary findings. Front. Psychol. 10:494. doi: 10.3389/fpsyg.2019.00494, PMID: 30890993 PMC6411849

[ref50] Lejeune F. Réveillon M. Monnier M. Hüppi P. S. Tolsa C. B. Barisnikov K. (2016). Social reasoning abilities in preterm and full-term children aged 5–7 years. Early Hum. Dev. 103, 49–54. doi: 10.1016/j.earlhumdev.2016.07.010, PMID: 27490664

[ref51] Linsell L. Malouf R. Morris J. Kurinczuk J. J. Marlow N. (2015). Prognostic factors for poor cognitive development in children born very preterm or with very low birth weight: a systematic review. JAMA Pediatr. 169, 1162–1172. doi: 10.1001/jamapediatrics.2015.2175, PMID: 26457641 PMC5122448

[ref52] Loewy J. (2015). NICU music therapy: song of kin as critical lullaby in research and practice. Ann. N. Y. Acad. Sci. 1337, 178–185. doi: 10.1111/nyas.12648, PMID: 25773633

[ref53] Lordier L. Loukas S. Grouiller F. Vollenweider A. Vasung L. Meskaldij D.-E.. (2019a). Music processing in preterm and full-term newborns: a psychophysiological interaction (PPI) approach in neonatal fMRI. NeuroImage 185, 857–864. doi: 10.1016/j.neuroimage.2018.03.078, PMID: 29630995

[ref54] Lordier L. Meskaldji D.-E. Grouiller F. Pittet M. P. Vollenweider A. Vasung L.. (2019b). Music in premature infants enhances high-level cognitive brain networks. Proc. Natl. Acad. Sci. 116, 12103–12108. doi: 10.1073/pnas.1817536116, PMID: 31138687 PMC6575179

[ref55] Loukas S. Lordier L. Meskaldji D. E. Filippa M. Sa de Almeida J. Van De Ville D.. (2022). Musical memories in newborns: a resting-state functional connectivity study. Hum. Brain Mapp. 43, 647–664. doi: 10.1002/hbm.25677 34738276 PMC8720188

[ref56] Månsson J. Källén K. Eklöf E. Serenius F. Ådén U. Stjernqvist K. (2021). The ability of Bayley-III scores to predict later intelligence in children born extremely preterm. Acta Paediatr. 110, 3030–3039. doi: 10.1111/apa.16037, PMID: 34289173

[ref57] Martinet M. Tolsa C. B. Jelidi M. R. Bullinger A. Perneger T. Pfister R. (2013). Élaboration et validation de contenu d’une grille d’observation du comportement sensorimoteur du nouveau-né à l’usage du personnel soignant. Arch. Pediatr. 20, 137–145. doi: 10.1016/j.arcped.2012.11.008, PMID: 23276600

[ref58] McGowan E. C. Vohr B. R. (2019). Impact of nonmedical factors on neurobehavior and language outcomes of preterm infants. NeoReviews 20, e372–e384. doi: 10.1542/neo.20-7-e372, PMID: 31261104

[ref59] Miller S. P. Ferriero D. M. Leonard C. Piecuch R. Glidden D. V. Partridge J. C.. (2005). Early brain injury in premature newborns detected with magnetic resonance imaging is associated with adverse early neurodevelopmental outcome. J. Pediatr. 147, 609–616. doi: 10.1016/j.jpeds.2005.06.033, PMID: 16291350

[ref60] Montagna A. Nosarti C. (2016). Socio-emotional development following very preterm birth: pathways to psychopathology. Front. Psychol. 7:169361. doi: 10.3389/fpsyg.2016.00080 PMC4751757 26903895

[ref61] Palazzi A. Nunes C. C. Piccinini C. A. (2018). Music therapy and musical stimulation in the context of prematurity: a narrative literature review from 2010–2015. J. Clin. Nurs. 27, e1–e20. doi: 10.1111/jocn.13893, PMID: 28544065

[ref62] Pierrat V. Marchand-Martin L. Arnaud C. Kaminski M. Resche-Rigon M. Lebeaux C.. (2017). Neurodevelopmental outcome at 2 years for preterm children born at 22 to 34 weeks’ gestation in France in 2011: EPIPAGE-2 cohort study. BMJ 358:j3448. doi: 10.1136/bmj.j3448 28814566 PMC5558213

[ref63] Pittet-Metrailler M. P. Mürner-Lavanchy I. M. Adams M. Bickle-Graz M. Pfister R. E. Natalucci G.. (2019). Neurodevelopmental outcome at early school age in a Swiss national cohort of very preterm children. Swiss Med. Wkly. 149:w20084. doi: 10.4414/smw.2019.20084 31154661

[ref64] Putnam S. P. Gartstein M. A. Rothbart M. K. (2006). Measurement of fine-grained aspects of toddler temperament: the early childhood behavior questionnaire. Infant Behav. Dev. 29, 386–401. doi: 10.1016/j.infbeh.2006.01.004, PMID: 17138293 PMC4334385

[ref65] Putnam S. P. Rothbart M. K. (2006). Development of short and very short forms of the Children's behavior questionnaire. J. Pers. Assess. 87, 102–112. doi: 10.1207/s15327752jpa8701_09, PMID: 16856791

[ref66] Réveillon M. Hüppi P. S. Barisnikov K. (2018). Inhibition difficulties in preterm children: developmental delay or persistent deficit? Child Neuropsychol. 24, 734–762. doi: 10.1080/09297049.2017.1294665 28279131

[ref67] Ritchie K. Bora S. Woodward L. J. (2015). Social development of children born very preterm: a systematic review. Dev. Med. Child Neurol. 57, 899–918. doi: 10.1111/dmcn.12783, PMID: 25914112

[ref68] Rothbart M. K. (1981). Measurement of temperament in infancy. Child Dev. 52, 569–578. doi: 10.2307/1129176

[ref69] Skiöld B. Alexandrou G. Padilla N. Blennow M. Vollmer B. Ådén U. (2014). Sex differences in outcome and associations with neonatal brain morphology in extremely preterm children. J. Pediatr. 164, 1012–1018. doi: 10.1016/j.jpeds.2013.12.051, PMID: 24530122

[ref70] Stephens R. L. Elsayed H. E. Reznick J. S. Crais E. R. Watson L. R. (2021). Infant attentional behaviors are associated with ADHD symptomatology and executive function in early childhood. J. Atten. Disord. 25, 1908–1918. doi: 10.1177/1087054720945019, PMID: 32749184 PMC8427808

[ref71] Symington A. J. Pinelli J. (2006). Developmental care for promoting development and preventing morbidity in preterm infants. Cochrane Database Syst. 2009. doi: 10.1002/14651858.CD001814.pub2 PMC8962209 16625548

[ref72] Taylor R. L. Rogers C. E. Smyser C. D. Barch D. M. (2023). Associations between preterm birth, inhibitory control-implicated brain regions and tracts, and inhibitory control task performance in children: consideration of socioeconomic context. Child Psychiatry Hum. Dev., 1–15. doi: 10.1007/s10578-023-01531-y PMC10949152 37119410

[ref73] Tooley U. A. Bassett D. S. Mackey A. P. (2021). Environmental influences on the pace of brain development. Nat. Rev. Neurosci. 22, 372–384. doi: 10.1038/s41583-021-00457-5, PMID: 33911229 PMC8081006

[ref74] Treyvaud K. Lee K. J. Doyle L. W. Anderson P. J. (2014). Very preterm birth influences parental mental health and family outcomes seven years after birth. J. Pediatr. 164, 515–521. doi: 10.1016/j.jpeds.2013.11.001, PMID: 24359937 PMC3950307

[ref75] Treyvaud K. Ure A. Doyle L. W. Lee K. J. Rogers C. E. Kidokoro H.. (2013). Psychiatric outcomes at age seven for very preterm children: rates and predictors. J. Child Psychol. Psychiatry 54, 772–779. doi: 10.1111/jcpp.12040, PMID: 23347471 PMC3821531

[ref76] Turpin H. Urben S. Ansermet F. Borghini A. Murray M. M. Müller-Nix C. (2019). The interplay between prematurity, maternal stress and children’s intelligence quotient at age 11: a longitudinal study. Sci. Rep. 9:450. doi: 10.1038/s41598-018-36465-2, PMID: 30679588 PMC6345959

[ref77] Twilhaar E. S. Wade R. M. De Kieviet J. F. Van Goudoever J. B. Van Elburg R. M. Oosterlaan J. (2018). Cognitive outcomes of children born extremely or very preterm since the 1990s and associated risk factors: a meta-analysis and meta-regression. JAMA Pediatr. 172, 361–367. doi: 10.1001/jamapediatrics.2017.5323, PMID: 29459939 PMC5875339

[ref78] Van der Heijden M. J. Oliai Araghi S. Jeekel J. Reiss I. K. M. Hunink M. M. Van Dijk M. (2016). Do hospitalized premature infants benefit from music interventions? A systematic review of randomized controlled trials. PLoS One 11:e0161848. doi: 10.1371/journal.pone.0161848, PMID: 27606900 PMC5015899

[ref79] Vasung L. Turk E. A. Ferradal S. L. Sutin J. Stout J. N. Ahtam B.. (2019). Exploring early human brain development with structural and physiological neuroimaging. NeuroImage 187, 226–254. doi: 10.1016/j.neuroimage.2018.07.041, PMID: 30041061 PMC6537870

[ref80] Volpe J. J. (2019). Dysmaturation of premature brain: importance, cellular mechanisms, and potential interventions. Pediatr. Neurol. 95, 42–66. doi: 10.1016/j.pediatrneurol.2019.02.016, PMID: 30975474

[ref81] Webb A. R. Heller H. T. Benson C. B. Lahav A. (2015). Mother’s voice and heartbeat sounds elicit auditory plasticity in the human brain before full gestation. Proc. Natl. Acad. Sci. 112, 3152–3157. doi: 10.1073/pnas.1414924112, PMID: 25713382 PMC4364233

[ref82] Westrup B. (2016). It is important with developmental supportive interventions beyond the NICU period. Acta Paediatrica, 105, 732–733. 27272627 10.1111/apa.13475

[ref83] Wildgruber D. Riecker A. Hertrich I. Erb M. Grodd W. Ethofer T.. (2005). Identification of emotional intonation evaluated by fMRI. Neuroimage 24, 1233–1241. doi: 10.1016/j.neuroimage.2004.10.034, PMID: 15670701

[ref84] Witt A. Theurel A. Tolsa C. B. Lejeune F. Fernandes L. de Jonge L.. (2014). Emotional and effortful control abilities in 42-month-old very preterm and full-term children. Early Hum. Dev. 90, 565–569. doi: 10.1016/j.earlhumdev.2014.07.008, PMID: 25105752

[ref85] Wood N. Costeloe K. Gibson A. Hennessy E. Marlow N. Wilkinson A. (2005). The EPICure study: associations and antecedents of neurological and developmental disability at 30 months of age following extremely preterm birth. Arch Dis Child Fetal Neonatal Ed 90, F134–F140. doi: 10.1136/adc.2004.052407, PMID: 15724037 PMC1721849

[ref86] Yue W. Han X. Luo J. Zeng Z. Yang M. (2021). Effect of music therapy on preterm infants in neonatal intensive care unit: systematic review and meta-analysis of randomized controlled trials. J. Adv. Nurs. 77, 635–652. doi: 10.1111/jan.14630, PMID: 33200833

